# Identification of a Novel Series of Potent TrkA Receptor Tyrosine Kinase Inhibitors

**DOI:** 10.1155/2012/412614

**Published:** 2012-05-02

**Authors:** Stéphane L. Raeppel, Frédéric Gaudette, Hannah Nguyen, Normand Beaulieu, James Wang, Christiane Maroun, Jeffrey M. Besterman, Arkadii Vaisburg

**Affiliations:** ^1^Department of Medicinal Chemistry, MethylGene Inc., 7150 Rue Frederick-Banting, Suite 200, Montréal, QC, Canada H4S 2A1; ^2^Department of Cell Biology and Pharmacology, MethylGene Inc., 7150 Rue Frederick-Banting, Suite 200, Montréal, QC, Canada H4S 2A1; ^3^Department of PK/Analytical Chemistry, MethylGene Inc., 7150 Rue Frederick-Banting, Suite 200, Montréal, QC, Canada H4S 2A1

## Abstract

A novel series of *N*-(3-(6-substituted-aminopyridin-3-yloxy)phenyl)-2-oxo-3-phenylimidazolidine-1-carboxamides targeting TrkA receptor tyrosine kinase was identified. SAR study of the series allowed us to design and synthesize compounds possessing inhibitory activity of TrkA kinase enzyme in the low nanomolar range with low residual activity against c-Met and with no significant activity against VEGFR2.

Tropomyosin-related kinases (Trks) are receptor tyrosine kinases normally expressed in neuronal tissue where they play important role in both development and function of the nervous system [1]. The Trk receptor family is composed of three members (A, B, and C) activated by specific ligands called neurotrophins (NT). Each Trk receptor contains an extracellular domain (ligand binding), a transmembrane region, and an intracellular domain (including kinase domain) which upon binding of their respective ligand (nerve growth factor (NGF) for TrkA, brain-derived growth factor (BDNF) and NT-4/5 for TrkB, and NT3 for TrkC) triggers oligomerization of the receptors, phosphorylation of specific tyrosine residues in the kinase domain, and downstream signal transduction pathways, including survival, proliferation, and differentiation in normal and neoplastic neuronal cells [2–4]. While Trks are expressed at low levels outside the nervous system in the adult, deregulation of TrkA and TrkB and their cognate ligands has been described in numerous types of cancers including prostate, breast, colorectal, ovarian, lung, pancreas, melanoma, thyroid, and neuroblastoma and occurs mainly through wild type receptor overexpression, activation, amplification, and/or mutation [5, 6]. Importantly, increased Trks activation in tumor tissues correlates with an aggressive phenotype and poor clinical outcome [6].

There are a limited number of reported selective TrkA receptor tyrosine kinase inhibitors in the literature (Figure 1) [7–9]. Developed by Cephalon (now a group member of Teva Pharmaceutical Industries Ltd.), lestaurtinib (**CEP-701**) is a potent multitargeted tyrosine kinases inhibitor targeting mainly TrkA, Flt3, and JAK2 and is in clinical trials for the treatment of myeloproliferative disorders [10, 11]. More recently, Cephalon disclosed indenopyrrolocarbazole **12a** as a potent and selective TrkA inhibitor displaying antitumor properties [12]. Moreover, organoruthenium-pyridocarbazole (**R**_**Ru**_)-21 from Pagano et al. [13], oxindole **3** from GSK [14], isothiazole **5n** from Pfizer [15], thiazole **20h** from BMS [16], and **AZ-23** from AstraZeneca were reported as potent and selective TrkA inhibitors as well [17, 18].

We have previously reported on four novel series of kinase inhibitors with a 2-oxo-3-phenylimidazolidine-1-carboxamide head group based on thieno[3,2-*b*]pyridine [19], quinoline [20], pyrimidine, and pyridine scaffolds [21], exemplified by structures **1–4**, respectively (Table 1). Bi-cyclic RTK inhibitors **1** and **2** strongly inhibited both c-Met and VEGFR2 enzymes while compounds **3** and **4** showed some preference for the c-Met kinase enzyme. In our efforts to identify new kinase inhibitors with a different kinase inhibitory profile from the ones of compounds **1–4** (and their analogues), we viewed compound **4** as a valuable starting point for further exploration. As the first exercise, we changed the substitution pattern between the central phenyl ring and the pyridine core of compound **4** as illustrated in Figure 2 (transposition from 1,4- to 1,3-substitution pattern like in isothiazole **5n** from Pfizer, thiazole **20h** from BMS, and pyrimidine **AZ-23** from AstraZeneca), thus synthesizing compound **5**. This compound was roughly ten times weaker than the parent compound **4** against both c-Met and VEGFR2 enzymes, but surprisingly showed potent inhibition of TrkA when tested at 0.1 *μ*M. As a consequence, we initiated the SAR study around compound **5** as a prototype of selective TrkA inhibitors. The IC_50_ of compound **5** was determined to be 4 nM against the TrkA human enzyme.

A straightforward synthesis of the target molecule **5** was developed and is illustrated in Scheme 1. (The details for the synthesis and the characterization of all the new compounds are well described in [21].) 5-Bromo-2-nitropyridine was reacted with 3-aminophenol in the presence of cesium carbonate in acetonitrile at room temperature. The choice of the base, solvent, and temperature is critical in order to obtain the intermediate **A** in fairly good yield; otherwise multiple products are formed. Intermediate **A** was subsequently reacted with 2-oxo-3-phenylimidazolidine-1-carbonyl chloride generated *in situ* from the reaction between 1-phenylimidazolidin-2-one and triphosgene to afford intermediate **B**. After reduction of the nitro group and subsequent derivatization of 6-aminopyridine intermediate **C**, compound **5** was obtained in good yield.

To continue with the SAR study around compound **5**, we explored the replacement of the oxygen linker by keeping unchanged the 2-oxo-3-phenylimidazolidine-1-carboxamide head group, which is presumed to occupy the hydrophobic back pocket of the kinase catalytic domain, and 6-acetamidopyridine scaffold, which is supposed to interact with the ATP catalytic site in the hinge region of the target kinase enzyme (Table 2). Not surprisingly, the replacement of the oxygen linker in compound **5** by an ureido, ethynyl, sulfamoyl, or carbamoyl spacer (compounds **6–9**) completely abolished the activity against TrkA kinase enzyme.

We next investigated the presence of a small substituent located on the central phenyl ring (positions 2 and 4) of compound **5** (Table 3). In contrast with either thiazole **20h** from Bristol-Myers Squib [16] or **AZ-23** from AstraZeneca [17, 18] (Figure 1), in which the presence of a small substituent on the central phenyl or pyridinyl ring improves the activity and the selectivity against the TrkA kinase enzyme, in our series of compounds that trend was not observed. Thus, compounds **10–12** turned out to be inactive against TrkA.

We finally examined the influence of each extremity of compound **5** where R^1^ is presumed to extend into the solvent exposed area and R^2^ is thought to occupy the hydrophobic back pocket of the kinase catalytic domain—both sites potentially capable of modulating physicochemical and pharmacokinetic properties of the inhibitors (compounds **13–17,** Table 4). Removal of the acetyl group from compound **5** afforded compound **13** with a lower potency against TrkA, probably due to the absence of a strong hydrogen bond donor (NH amide) and/or a hydrogen bond acceptor (carbonyl amide) which is presumed to interact with the hinge region of the kinase binding domain. Introduction of relatively small electronegative fluorine in the *para* position of the phenyl ring is well tolerated (compound **14**). Finally, replacement of the acetamido group by a functionalized ureido moiety is also accepted, giving rise to potent TrkA inhibitors (compounds **15–17**) and allowing us to incorporate water solubilizing groups.

Compound **5** was profiled and tested at 100 nM against 24 other kinase enzymes using Millipore's Kinaseprofiler assay services. In addition to targeting TrkA receptor tyrosine kinase (98% inhibition), it was also moderately active against Flt3 (83% inhibition) but, importantly, did not show significant inhibitory activity against the other representative human kinases (<40% inhibition): ALK, Bmx, CHK1, cKit, c-Raf, EphB4, FAK, GSK3*β*, Haspin, IKK*β*, JAK2, JAK3, LIMK1, MEK1, c-Met, PDK1, PI3-Kinase*β*, Pim-1, PKB*α*, Ret, Ron, and Tie2. When tested at 10 nM against TrkB, compound **5** showed 40% inhibition.

Compound **5** was further evaluated for its pharmacokinetic properties in the rat (Table 5). Compound **5 **showed a 1.5 h half-life, a high rate of clearance, an appreciable steady-state volume of distribution, and excellent oral bioavailability.

In conclusion, a novel series of *N*-(3-(6-substituted-aminopyridin-3-yloxy)phenyl)-2-oxo-3-phenylimidazolidine-1-carboxamides was designed and synthesized. The most attractive compounds of the series—**5** and **14–17**—are selective for the TrkA kinase enzyme in the low nanomolar range (comparable with the known TrkA inhibitors) with only residual activity against other RTKs. These novel chemical entities represent a valuable starting point in design and synthesis of TrkA selective inhibitors both as tools to further validate TrkA as a promising biological target and for the development of potential new anticancer therapies.

## Figures and Tables

**Figure 1:**
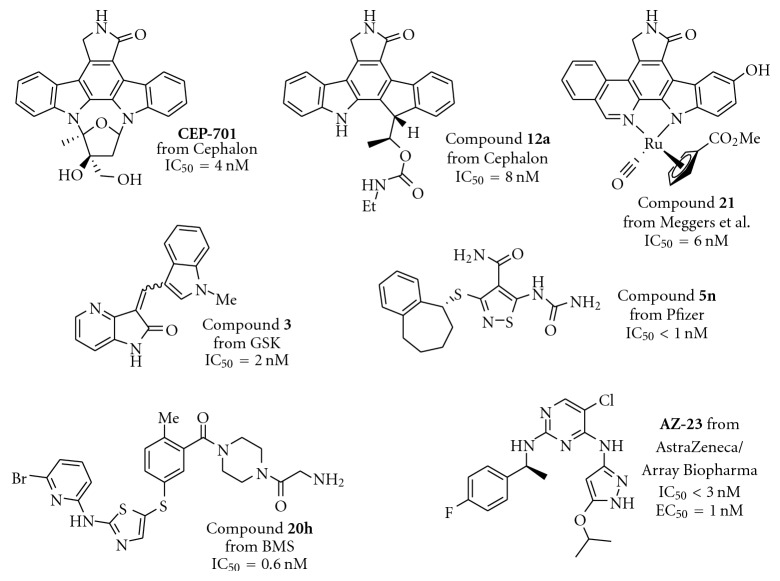
TrkA inhibitors disclosed in the literature and their reported IC_50_ and/or EC_50_.

**Figure 2:**
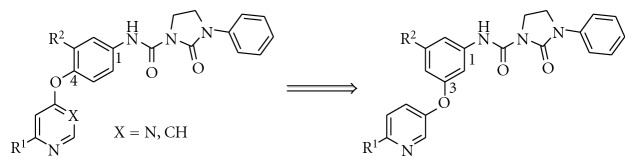
Transposition from 1,4- into 1,3-substitution pattern for the central phenyl ring.

**Scheme 1:**
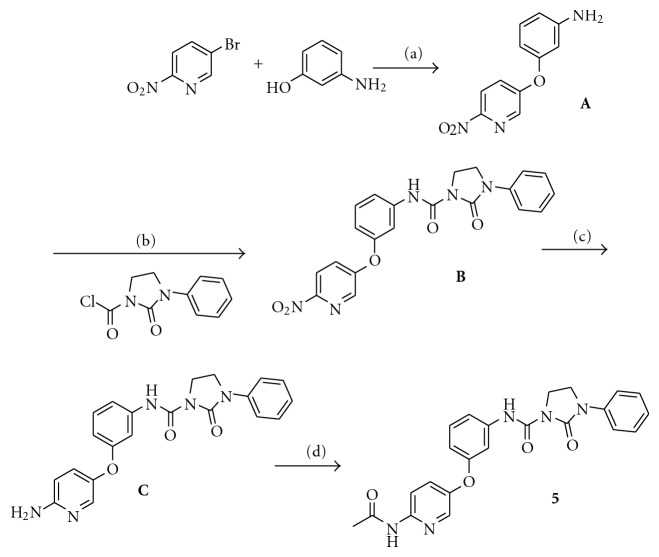
Synthesis of compound **5**: (a) Cs_2_CO_3_, MeCN, rt; (b) DIPEA, DCM, rt; (c) iron, NH_4_Cl, MeOH/water, reflux; (d) Ac_2_O, rt.

**Table 1:** 2-Oxo-3-phenylimidazolidine-1-carboxamide kinase inhibitors based on different scaffolds.

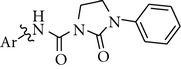				
Cpd	Ar	c-Met^a^ IC_50_ (*μ*M)	VEGFR2^a^ IC_50_ (*μ*M)	TrkA^a^ % inh. at 0.1 *μ*M

**1**	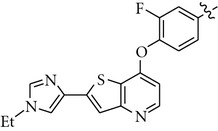	0.02	0.01	82
**2**	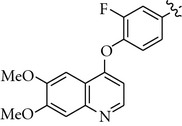	0.03	0.01	94
**3**	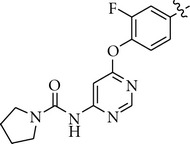	0.05	0.15	16
**4**	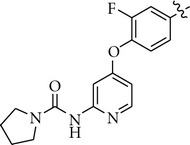	0.03	0.23	49
**5**	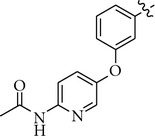	0.31	2.50	98

^a^IC_50_ and percentage of inhibition values are reported as the average of ≥3 experiments.

**Table 2:** Influence of the linker in the 6-acetamidopyridine series on the TrkA potency.

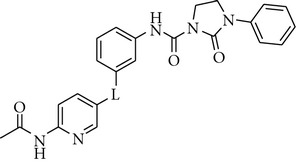		
Cpd	L	TrkA^a^ % inh. at 30 nM

**5**	O	96
**6**	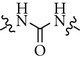	5
**7**	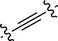	16
**8**	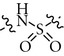	6
**9**	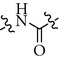	2

^a^percentage of inhibition values are reported as the average of ≥3 experiments.

**Table 3:** Influence of the central phenyl ring substistution in the 6-acetamidopyridine series on the TrkA potency.

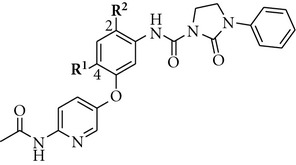			
Cpd	**R** ^1^	**R** ^2^	TrkA^a^ % inh. at 30 nM

**5**	H	H	96
**10**	Me	H	6
**11**	OMe	H	15
**12**	H	Cl	0

^a^percentage of inhibition values are reported as the average of ≥3 experiments.

**Table 4:** Influence of **R^1^** and **R^2^** substituents on the TrkA potency.

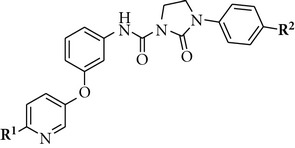			
Cpd	**R** ^1^	**R** ^2^	TrkA IC_50_ (nM)

**5**	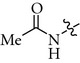	H	4
**13**	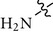	H	20
**14**	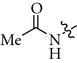	F	3
**15**	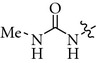	H	5
**16**	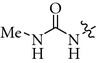	F	2
**17**	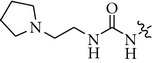	F	4

^a^IC_50_ values are reported as the average of ≥2 experiments.

**Table 5:** Rat pharmacokinetic profile of compound **5**.

PK properties^a^	Cpd **5**
T_1/2_ (h), i.v.	1.5
Cl (L/h/kg) i.v.	3.3
*V*ss (L/kg) i.v.	3.7
*t*_max_ (h), p.o.	4.0
*N*. *C*_max_ [*μ*M/(mg/kg)], p.o.	0.08
*N*. AUC [*μ*M.h/(mg/kg)], p.o.	0.83
*F* (%)	99

^a^i.v. dose: 2.58 mg/kg; p.o. dose: 5.25 mg/kg.
